# Socio-cultural determinants of adiposity and physical activity in preschool children: A cross-sectional study

**DOI:** 10.1186/1471-2458-10-733

**Published:** 2010-11-26

**Authors:** Flavia Bürgi, Ursina Meyer, Iris Niederer, Vincent Ebenegger, Pedro Marques-Vidal, Urs Granacher, Susi Kriemler, Jardena J Puder

**Affiliations:** 1 Institute of Exercise and Health Sciences, University of Basel, Birsstrasse 320B, 4052 Basel, Switzerland; 2 Institute of Sports Sciences and Physical Education, University of Lausanne, Bâtiment de Vidy, 1015 Lausanne, Switzerland; 3 Institute of Social and Preventive Medicine, Centre Hospitalier Universitaire Vaudois and Faculty of biology and medicine, University of Lausanne, Rue de Bugnon 17, 1005 Lausanne, Switzerland; 4 Swiss Tropical and Public Health Institute, University of Basel, Socinstrasse 57, 4002 Basel, Switzerland; 5 Service of Endocrinology, Diabetes and Metabolism, Centre Hospitalier Universitaire Vaudois, University of Lausanne, Rue de Bugnon 46, 1011 Lausanne, Switzerland

## Abstract

**Background:**

Both individual socio-cultural determinants such as selected parental characteristics (migrant background, low educational level and workload) as well as the regional environment are related to childhood overweight and physical activity (PA). The purpose of the study was to compare the impact of distinct socio-cultural determinants such as the regional environment and selected parental characteristics on adiposity, PA and motor skills in preschool children.

**Methods:**

Forty preschools *(N *= 542 children) of two culturally different urban regions (German and French speaking part of Switzerland) participated in the study (Ballabeina Study). Outcome measures included adiposity (BMI and skinfold thickness), objectively measured sedentary activities and PA (accelerometers) and agility performance (obstacle course). Parental characteristics (migrant status, educational level and workload) were assessed by questionnaire.

**Results:**

Children from the French speaking areas had higher adiposity, lower levels of total and of more intense PA, were more sedentary and less agile than children from the German speaking regions (percent differences for all outcome parameters except for BMI ≥10%; all *p *≤ 0.04). Differences in skinfold thickness, sedentary activities and agility, but not in PA, were also found between children of Swiss and migrant parents, though they were ≤8% (*p *≤ 0.02). While paternal workload had no effect, maternal workload and parental education resulted in differences in some PA measures and/or agility performance (percent differences in both: ≤9%, *p *≤ 0.008), but not in adiposity or sedentary activities (*p *= NS). Regional differences in skinfold thickness, PA, sedentary activities and agility performance persisted after adjustment for parental socio-cultural characteristics, parental BMI and, where applicable, children's skinfolds (all *p *≤ 0.01).

**Conclusions:**

The regional environment, especially the broader social environment, plays a prominent role in determining adiposity, PA and motor skills of young children and should be implicated in the prevention of obesity and promotion of PA in children.

**Trial Registration:**

clinicaltrials.gov NCT00674544

## Background

Childhood overweight and obesity have been increasing dramatically worldwide, even in young children. Despite a possible stabilization, the high prevalence remains a great public health concern [1]. Among several environmental factors, a sedentary behaviour (especially TV viewing) and a reduction in physical activity (PA) are implicated in this increase in body fatness [2]. Some data indicate that children have become less physically active [3-5]. One potential environmental influence on children's PA that may be implicated in this decrease is the amount of time spent outdoors [6]. It has recently been reported that 3- to 5-year old children spend around 80% of their time in activities classified as sedentary or at most light PA [7]. Furthermore, a trend towards a decline in motor performance has already been noticed in young children [8].

Individual socio-cultural determinants such as selected parental characteristics (migrant background, low educational level and high workload) are known risk factors for childhood overweight/obesity and sedentary behaviours [9-12]. In addition, there exist also regional variations of overweight and PA [13,14]. Even within Europe, prevalence in overweight differs among countries, ranging from 5 to 25% [13,14]. However, there is a lack of data on the respective roles of individual and environmental determinants in a well defined setting. With its linguistic and cultural diversity, Switzerland offers the opportunity to study diverse cultural environments within the same country. It can thus serve as a model to examine the impact of the regional environment on adiposity, PA and motor skills in children.

In the present study, we assessed differences in adiposity, objectively measured PA, sedentary behaviour and agility performance in preschool children according to different socio-cultural determinants (parental migrant status, education, and workload) and the regional environment.

## Methods

### Study design and participants

All children participated in the Ballabeina-Study, the description of which has been reported previously [15]. Briefly, 40 public preschools with a high migrant prevalence (>40% migrants) were randomly selected in two distinct similar-sized urban areas at the north-eastern and south-western edge of Switzerland, respectively. Of note, the majority of preschools in Switzerland are public. Twenty schools were located in the German speaking region (north-eastern part of Switzerland; city of St. Gallen: 70'000 inhabitants) and 20 in the French speaking region (south-western part of Switzerland; urban surroundings of Lausanne: 50'000 inhabitants). The study was approved by both cantonal ethical committees and the parents or legal representatives of each child provided written informed consent. Of the initial 727 preschool children, informed consent was obtained from 655 (participation rate: 90.1%) and valid PA measures were obtained from 542 children (83% of the participating children). This data set was used for further analyses. Children without valid PA data had higher BMI (16.0 ± SD 1.9 vs. 15.6 ± SD 1.4, *p = *0.01), but did not differ in body fat, agility performance, migrant status and parental education compared to children with valid PA-data (*p *> 0.20). Of those with valid PA measures, complete questionnaire data were obtained from 485 children (74% of the participating children).

### Adiposity

Body height and weight were measured by standardized procedures [15]. Body mass index (BMI) was calculated by dividing weight by height squared. Prevalence of overweight/obese children was calculated according to IOTF criteria [16]. We used the sum of 4 skinfold thicknesses (triceps, biceps, subscapular and suprailiac) as indicator of body fat. Skinfold thicknesses were measured with a Harpenden caliper using standard procedures [17].

### Physical activity and sedentary activities

PA was measured over 6 consecutive days with an accelerometer (GT1M, Actigraph, Florida, USA), worn around the hip and programmed to save data in 15-second intervals (epochs). The Actigraph/CSA is the most common used motion sensor in children and has a good reproducibility, validity and feasibility [18]. This type of PA assessment has been shown to be valid across different activities in 3- to 5-year old children, with a Pearson correlation coefficient between VO_2 _(ml/kg per min) and epochs of *r *= 0.82 [19].

To consider data as valid, at least 3 days of recording (2 weekdays and 1 weekend day) [20] with a minimum of 6 h registration per day were needed. The 6-h-validity was highly correlated with 10-h-validity (*N *= 502, *r *= 0.92, *p *< 0.001). Data from monitored days were extrapolated by weighing weekdays and weekends (5:2). Sequences of at least 10 minutes of consecutive zero values were removed and interpreted as "accelerometer not worn" [21].

Total PA, moderate-vigorous physical activity (MVPA) and vigorous physical activity (VPA) were chosen as markers of PA. Total PA was expressed as counts per minute (cpm, total counts recorded divided by daily wearing time). MVPA and VPA were based on cut-offs published by Pate et al. [19]: ≥420 counts/15 s for MVPA and ≥842 counts/15 s for VPA. Sedentary activities were defined as 0-25 counts/15 s [22]. Each 15-second-interval over or under the specific cut-off was summarized in the corresponding intensity level group and data are presented as the amount of 15-second-intervals per day. Differences in daily wearing time between the groups were negligible. Therefore, we did not adjust for this variable (mean wearing time 10.8 h/day).

### Motor skills

Agility was chosen as a measure of motor skills [23] and was assessed by the time needed to complete an obstacle course. This test was described by Vogt and Kunz [24,25] for 3- to 6-year old children. It includes running 1 m from a marking cone to a transversally positioned bench, jumping over the bench (36 cm high, 28 cm wide), crawling under the bench and running back to the marking cone three times in a row as fast as possible. Time was measured in seconds. Each child had two attempts and the faster trial was used for further data analysis. The intra- and interobserver correlations in our pilot study (*n *= 14) were *r *= 0.99 *(p *< 0.01) and *r *= 0.82 (*p *< 0.01), respectively.

### Socio-cultural determinants

We investigated the respective role of different socio-cultural determinants: the broad regional environment on one side and individual socio-cultural determinants, such as parental migrant status, educational level and workload (parental characteristics) on the other side. The regional socio-cultural environment was defined by language and geographical region (German speaking, north-eastern versus French speaking south-western part of Switzerland) [26]. Both regions have a very similar climate. For information about parental socio-cultural characteristics (migrant status, education and workload), parents filled out a general health questionnaire. Parental migrant status was determined by their country of birth (e.g. born outside of Switzerland) the educational level as the respective highest grade of school completed (5 levels). Low educational level was defined as no education beyond mandatory school (9 years). For analyses, migrant status and low parental education were divided in two categories (at least one parent migrant/with low education - no parent migrant/with low education). Maternal and paternal workloads were divided into three categories: no professional activity, part-time and full-time. Due to school legislation, no information could be obtained about economic (i.e. earning, wages) data.

Time spent playing outdoors (min/day) as an additional measure of PA [6] and television (TV) time (min/day) as an additional measure of sedentary behaviour were also asked to the parents. The questionnaire also asked for ways of commuting to school and barriers to play outdoors such as road traffic, lack of playgrounds or courtyards, danger of crime or simply the lack of interest to play outdoors.

### Statistical analyses

All analyses were performed using STATA version 11.0 (Statacorp, College Station, Tx, USA). Differences in the subjects' characteristics between the 2 regions were compared using independent t-tests for continuous variables and Chi square-calculations for categorical variable. Measures of adiposity, PA and agility performance are presented as means ± standard deviations (SD), as all variables were normally distributed. We used mixed linear or logistic regression models with adiposity measures, PA, sedentary behaviour and agility performance as outcome variables; regional environment or parental characteristics, as respective fixed factors and preschool class as random effect. Between-group differences were also expressed in percentages using the healthier behaviour (i.e. higher PA) or lower adiposity measure as a denominator. To test the independent effect of the regional environment (fixed factor), regression models were subsequently adjusted for sex, age, parental migrant status, educational level, workload and BMI & children's skinfolds. Significance was assumed at *p *< 0.05.

## Results

### Baseline characteristics

Baseline characteristics of the study population are presented in Table 1. Significant regional differences in parental socio-cultural factors were observed (all *p *< 0.05). In the French part, more parents were migrants, had a low education and a higher workload (all *p *< 0.05). For migrant parents, years living in Switzerland did not differ between the German and the French parts (*p *> 0.1). The most important migrant regions in both parts of Switzerland are shown in Table 1.

**Table 1:** Subjects' characteristics

	Total *n = *542	German speaking *n = *267	French speaking *n = *275	*p*-Value
Mean age (years)	5.1 ± 0.60	5.1 ± 0.59	5.2 ± 0.62	0.2
Sex: Boys/Girls %	264/278 49/51	131/136 49/51	133/142 48/52	0.8
Parental migrant status: migrant^1^/non-migrant %	391/151 72/28	176/91 66/34	215/60 78/22	< 0.001
Parental education: low^2^/middle, high %	179/306 37/63	68/165 29/71	111/141 45/55	< 0.001
Paternal workload: no/part time/full time %	17/24/445 3/5/92	7/9/216 3/4/93	10/15/229 4/6/90	0.5
Maternal workload: no/part time/full time %	182/213/99 37/43/20	88/117/28 38/50/12	94/96/71 36/37/27	< 0.001
Most frequent migrant countries		Balcan area 37% Asian countries 13% Germany 10%	Portugal 28% African countries 18% France 6%	

^1 ^at least 1 parent born outside Switzerland

^2 ^at least 1 parent with no education beyond mandatory school (9 year)

Data are shown as means +/- SD or as percentage

### Differences due to the regional environment

Differences in adiposity, PA measures and agility performance between the children in the German and French speaking part are shown in Table 2. BMI, skinfold thickness, sedentary activities and TV viewing time were higher and total PA, MVPA, VPA and agility performance were lower in the French speaking compared to the German speaking part (all *p *≤ 0.04)). Percent differences in all measures except for BMI were 10% or more.

**Table 2:** Differences in adiposity, PA and agility performance between the German and French speaking region

	German speaking (mean ± SD) *n = *267	French speaking (mean ± SD) *n = *277	*p*-Value
BMI (kg/m^2^)	15.5 ± 1.5	15.7 ± 1.4	0.04
Skinfold thickness (mm)	25.5 ± 7.8	28.2 ± 8.3	< 0.001
Percentage overweight/obese children (%)	9.0	11.0	0.5
Total PA (counts per minute, cpm)	771 ± 169	684 ± 151	< 0.001
MVPA (number of 15-s-intervals/day)	400 ± 110	361 ± 101	0.001
VPA (number of 15-s-intervals/day)	110 ± 45	95 ± 42	0.006
Time spent playing outdoors (min/day)	110 ± 59	74 ± 47	< 0.001
Sedentary activities (number of 15-s-intervals/day)	1276 ± 216	1400 ± 253	< 0.001
TV time (min/day)	45 ± 43	67 ± 50	0.001
Obstacle course (s)	18.3 ± 3.3	20.0 ± 5.1	< 0.001

Variables were analyzed using mixed linear or logistic regression models with preschool class as a random effect. Data are shown as means +/- SD or as percentage.

When considering only children of migrant parents (*n *= 391), regional differences in all outcome parameters were similar to those observed in the total population (all *p *< 0.05). This was also true for children of Swiss parents (*n *= 151), except that the differences in BMI (*p *= 0.20), % body fat (*p *= 0.07) and agility performance (*p *= 0.1) were not significant. Finally, mentioned barriers to play outside (road traffic, lack of playgrounds or courtyards, danger of crime or lack of interest to play outdoors) did not differ between both regions (all *p *= NS).

### Differences due to parental characteristics

#### Migrant status

Compared to children of Swiss parents, children of migrant parents had higher skinfold thickness, were more sedentary, spent more time watching TV and were less agile (all *p *≤ 0.02, Table 3). Those differences generally ranged from 4-8%, while a stronger difference was found for time watching TV (75%). Conversely, there were no differences in BMI and PA.

**Table 3:** Differences in adiposity, PA and agility performance between children of migrant and non-migrant parents

	Non-migrants (mean ± SD) *n = *151	Migrants^**1 **^(mean ± SD) *n = *393	*p*-Value
BMI (kg/m^2^)	15.5 ± 1.1	15.7 ± 1.5	0.2
Skinfold thickness(mm)	25.3 ± 5.7	27.4 ± 8.9	0.01
Percentage overweight/obese children (%)	7.0	11.0	0.1
Total PA (counts per minute, cpm)	752 ± 176	717 ± 161	0.2
MVPA (number of 15-s-intervals/day)	379 ± 103	380 ± 109	0.5
VPA (number of 15-s-intervals/day)	104 ± 45	102 ± 44	0.8
Time spent playing outdoor (min/day)	99 ± 58	86 ± 54	0.1
Sedentary activities (number of 15-s-intervals/day)	1291 ± 229	1357 ± 247	0.02
TV time (min/day)	34 ± 30	65 ± 48	< 0.001
Obstacle course (s)	18.2 ± 3.5	19.5 ± 4.7	0.005

^1^at least one parent born outside Switzerland

Variables were analyzed using mixed linear or logistic regression models with preschool class as a random effect. Data are shown as means +/- SD or as percentage.

#### Parental educational level

No significant differences were found in adiposity or measured PA according to the parental educational level (low vs. middle/high). However, children of parents with low education were less agile (mean difference: 1.5 s, *p *< 0.001) and watched more TV (mean difference: 33 min, *p *< 0.001) than children of parents with middle/high educational level. Differences in agility amounted to 8%, but a stronger difference was found for time watching TV (75%). Similar results were obtained when maternal and paternal educational levels were analyzed separately according to 5 educational levels (data not shown).

#### Parental workload

Nine out of ten (92%) fathers worked fulltime. Paternal workload was not related to differences in adiposity, PA and agility performance of the children. Conversely, compared to children of mothers without professional activity, children of part-time working mothers had higher total PA (mean difference: 45 cpm, *p *= 0.006) and spent more time in VPA (mean difference: 10 intervals of 15 s, *p *= 0.02), were more agile (mean difference: -1.2 s, *p *= 0.008) and watched less TV (mean difference: 10 min, *p *= 0.03), while children of full-time working mothers were more sedentary (mean difference: 61 intervals of 15 s, *p *= 0.04). Children of working mothers (both part-time and full-time) spent more time in MVPA than children of mothers without professional activity (mean difference: 25 resp. 26 intervals of 15 s, *p *= 0.02 resp. *p *= 0.046). Altogether, these differences amounted to ≤9%, while a large difference was found for time watching TV (20%).

### Effect of the regional environment

Effect sizes of differences between the French compared to the German region are presented in Table 4 before and after adjustment. Regional differences in body fat, PA, sedentary activities and agility performance persisted after adjustment for sex, age, parental socio-cultural characteristics, parental BMI and children's skinfolds. Only differences in BMI did not remain significant (*p *= 0.3). Adjusting only for parental characteristics (either analyzing migrant status, educational level and workload separately or together) did not alter these results (data not shown). On the other hand, only the following differences according to parental characteristics remained significant: higher time spent watching TV (children of migrant and low educational level parents), higher adiposity (children of migrant parents) and a higher total PA and MVPA (children of part-time working mothers compared to children of mothers without professional activity); all *p *≤ 0.03. In most circumstances, their effect was attenuated compared to the non-adjusted values (data not shown).

**Table 4:** Multivariate-adjusted regression model analyzing differences of the French compared to the German speaking region in Switzerland

Outcome variable	Crude model: Adjusted for sex & age		Adjusted Model: Adjusted for sex, age, parental characteristics and adiposity	
	**β-coefficient (95% CI)**	***p*-value**	**β-coefficient (95% CI)**	***p*-value**
BMI	0.3 (0.02; 0.5)	0.03	0.2 (-0.1; 0.4) ^1^	0.3
Skinfolds (mm)	2.7 (1.3; 4.1)	< 0.001	2.1 (0.4; 3.7) ^1^	0.01
Total PA (cpm)	-88 (-127; -50)	< 0.001	-99 (-133; -64)	< 0.001
MVPA (15-s-intervals/day)	-52 (-77; -27)	0.001	-52 (-73; -31)	< 0.001
VPA (15-s-intervals/day)	-15 (-26; -5)	0.004	-18 (-29; -7)	0.001
Time spent outdoors (min/day)	-35 (-48; -21)	< 0.001	-30 (-44; -16)	< 0.001
Sedentary activities (15-s-intervals/day)	125 (85; 165)	< 0.001	134 (85; 182)	< 0.001
TV time (min/day)	22 (10; 34)	< 0.001	16 (4; 28)	0.01
Obstacle course (s)	1.8 (1.0; 2.6)	< 0.001	1.4 (0.6; 2.3)	0.001

^1 ^adjustment was done without children's skinfolds.

Variables were analyzed using mixed linear regression models with preschool class as a random effect with adjustment for sex and age (crude model) or for sex, age, parental migrant status, educational level, workload and BMI & children's skinfolds (adjusted model). Results are expressed as β-coefficient (95% CI).

## Discussion

Our results strengthen the importance of the regional environment on lifestyle behaviours and obesity in very young children. The main findings of this study are: Within the same country with the same national health policy, preschool children from the French speaking, south-western part of Switzerland had an increase in adiposity, were more sedentary, less physically active and less agile compared to the German speaking, north-eastern part. Those differences were in the range of 10% or more and persisted after adjustment for parental characteristics. On the other side, parental characteristics like migrant status, low educational level or workload had less impact on adiposity and lifestyle behaviour and their impact was attenuated after adjustment for the regional environment.

Children of migrant parents had more body fat, were more sedentary, less active, and less agile than children of Swiss parents. These findings are in agreement with several studies, which reported that children with a migration background had higher BMI and were less active [10,11,27,28]. Maternal workload and low parental educational level resulted in differences in some PA measures and/or in agility performance, but, in contrast to previous studies [10], not in adiposity. Ethnic and genetic factors can contribute to the increase in adiposity in some migrants, while economic reasons, lack of parental time and support and individual socio-cultural attitudes might also explain some of our data [29]. We could hypothesize that the healthy behaviour found in children of part-time working mothers could be a combination of time, motivation and sufficient financial resources. Parental characteristics had a particularly strong influence on the time spent watching TV. Existing differences in parental characteristics between the two regions could theoretically explain the observed differences in the regional environment. However, the impact of the regional environment on adiposity and all lifestyle behaviours except TV viewing was much larger and persisted after adjustment for the above mentioned parental characteristics.

The regional environment represents the broader social, cultural, economic and built environment, within which individual behaviour occurs [30]. Differences in the socio-cultural environment could explain our findings which are also in concordance with the observed North-South gradient in overweight and obesity within Europe [13,14]. In those studies, a particularly high prevalence of overweight/obesity was described among southern European countries [13,14]. This North-South gradient has also been reported within Italy [31]. Moreover, lower levels of overweight and obesity were found among children in countries of Central and Eastern Europe compared to Western Europe [13,14]. Different regional patterns have been observed for reported PA in children across European countries [13,32,33]. According to our results, the German speaking part of Switzerland reflects the trends in the north-eastern countries, whereas the French speaking region reflects those in the south-western (and Latin) European countries. Even within the same country, with almost identical climatic conditions, we could observe this North-South gradient in two urban areas. This could be explained by different prevailing cultural norms, priorities, attitudes and beliefs. Indeed, differences in health beliefs have been associated with differences in PA in young adults [33]. Furthermore, similarly to many northern countries, general efforts in health promotion have a stronger tradition in the north-eastern part of Switzerland compared to the south-western counterparts. Indeed, differences in health between the German and French speaking part of Switzerland have been shown to reflect broader European patterns [26]. Similar differences in PA within Switzerland were also observed in large representative samples of adults, where PA levels, reported by questionnaires, were found to be higher in citizens of the German compared to the French speaking part of Switzerland [26,34]. In addition, reported time spent playing outdoors has been shown to be higher in school-aged children and adolescents of the German compared to the French speaking part of Switzerland [35]. Similarly, substantial differences in "active transportation to school" have been documented between German- and French speaking school children and adolescents that were independent of socio-demographic characteristics and environmental factors like distance to school or bike availability [36]. On the other side, lifestyle behaviours in different regions within the French or within the German part of Switzerland, respectively, are reported to be very similar [26,34,35]. In our sample, the observed regional differences were comparable, if the whole population or the subgroup of non-migrant children were analyzed. On an even larger perspective outside of the health sector, differences between the German and French speaking part exist regarding educational and employment levels [37]. But also public transport and school system differ between the two parts and are possibly tightly linked to the key outcome measures of our study. For example, preschool in the French part of Switzerland resembles primary school more than it does in the German part of Switzerland (choice of academic objectives, classrooms).

A range of social and environmental factors have been described as potential influences on children's PA and sedentary behaviours [38]. The social environment includes social factors in the home and neighbourhood environments, as well as social peer networks. Within the social environment, the social network plays a major role. In adults, it can even influence adiposity more strongly than first grade relatives [39]. Since diverse phenomena can spread within social networks, it seems also possible that obesity or PA might spread from person to person [39]. Adults as well as children are embedded in social networks and are influenced by the evident behaviours of those around them. This fact suggests that weight gain or activity levels in one person might influence weight gain or activity levels in others. Having obese or sedentary social contacts might influence the adoption of such specific behaviours. In adolescent girls, the role of peer social network factors is crucial for participating in PA [40]. It is quite possible, that also in younger age not only home support and parental modelling, but also larger social peer networks play an important role [38].

The built environment has also been associated with PA and adiposity and includes access to recreation facilities, parks, playgrounds and traffic [30]. Our regional differences in the outcome parameters observed within the same country could also be influenced by differences in the built environment. Observed differences in time playing outdoors may reflect potential disparities in the access to playgrounds between the two regions, but might also be influenced by the social environment. In this age group, playing outdoors has been shown to be a good marker of PA [6]. Perceived barriers for children to play outdoors like road traffic, lack of playgrounds or courtyards and danger of crime did not differ between both regions, but the parental perception of barriers might be influenced by socio-cultural factors. Furthermore, ways of commuting and school-based structured PA as important factors of PA did not have a large impact on our results, as the great majority of preschoolers in both areas (90%) still walked to school and physical education at school did not differ.

Strengths and novelties of the study include the joint comparison of regional and individual determinants within the same small country taking advantage of two distinct socio-cultural regions of similar size and with a similar climate. Further strengths are the comprehensive assessment of adiposity and objectively measured PA and the inclusion of motor skills in a very young population. In this study, we focused on socio-cultural characteristics and therefore we did not take into account other essential factors that influence lifestyle and adiposity, such as parenting practices and believes. On the other side, parenting practices and believes may also be influenced by individual and regional socio-cultural characteristics. Since our investigation is not based on a representative sample, we cannot be certain to draw conclusions for the whole population, although differences in reported PA between the French and German regions in large representative samples of adults and in a population of school-aged children and adolescents confirm our findings [26,34-36]. Another limitation is the cross sectional design of the study that limits the investigation of clear cause-effect relationships.

## Conclusions

Differences in the regional and especially the broader social environment have a major impact in determining adiposity, sedentary behaviour, PA and motor skills of young children. Although the magnitude of their influence may depend on the specific lifestyle behaviour, their impact on adiposity, PA and motor skills may even exceed the influence of parental socio-cultural characteristics. Should our data be confirmed in future longitudinal studies, high-risk regions in addition to high-risk groups should be identified for future interventions. Thereby, a priority should be made for community-based programs that influence social norms and priorities and integrate also other sectors like the school and public transport system as well as the adaption of the environment. Specifically, this could include a raising awareness for the benefits of a healthy lifestyle behaviour and provision of opportunities for recreational activities and outdoor playing.

## Abbreviations

BMI: Body Mass Index; PA: physical activity; MVPA: moderate and vigorous physical activity; VPA: vigorous physical activity; cpm: counts per minute; TV: television;

## Competing interests

The authors declare that they have no competing interests.

## Authors' contributions

JJP and SK and designed the study. JJP was the principal investigator and is guarantor. JJP, SK, FB, IN, VE, UM, UG and PM established the methods and questionnaires. FB, IN, VE and JP were the main coordinators of the study. FB, IN, VE, UM, PM and JJP conducted the study. PM gave statistical and epidemiological support. FB wrote the article under the assistance of JJP and got additional help from SK, UG and PM. JJP obtained the funding, with the assistance of SK. All authors provided comments on the drafts and have read and approved the final version.

## Pre-publication history

The pre-publication history for this paper can be accessed here:

http://www.biomedcentral.com/1471-2458/10/733/prepub

## References

[B1] Ogden CL Carroll MD Curtin LR Lamb MM Flegal KM Prevalence of high body mass index in US children and adolescents, 2007-2008 JAMA 2010 303 3 242 249 10.1001/jama.2009.2012 20071470

[B2] Ekelund U Sardinha LB Anderssen SA Harro M Franks PW Brage S Cooper AR Andersen LB Riddoch C Froberg K Associations between objectively assessed physical activity and indicators of body fatness in 9- to 10-y-old European children: a population-based study from 4 distinct regions in Europe (the European Youth Heart Study) Am J Clin Nutr 2004 80 3 584 590 15321796 10.1093/ajcn/80.3.584

[B3] Martin M Dollman J Norton K Robertson I A decrease in the association between the physical activity patterns of Australian parents and their children; 1985-1997 J Sci Med Sport 2005 8 1 71 76 10.1016/S1440-2440(05)80026-X 15887903

[B4] Andersen LB van Mechelen W Are children of today less active than before and is their health in danger? What can we do? Scand J Med Sci Sports 2005 15 5 268 270 10.1111/j.1600-0838.2005.00488.x 16181249

[B5] Dollman J Norton K Norton L Evidence for secular trends in children's physical activity behaviour Br J Sports Med 2005 39 12 892 897 discussion 897 10.1136/bjsm.2004.016675 16306494 PMC1725088

[B6] Cleland V Crawford D Baur LA Hume C Timperio A Salmon J A prospective examination of children's time spent outdoors, objectively measured physical activity and overweight Int J Obes (Lond) 2008 32 11 1685 1693 10.1038/ijo.2008.171 18852701

[B7] Reilly JJ Jackson DM Montgomery C Kelly LA Slater C Grant S Paton JY Total energy expenditure and physical activity in young Scottish children: mixed longitudinal study Lancet 2004 363 9404 211 212 10.1016/S0140-6736(03)15331-7 14738795

[B8] Tomkinson GR Olds TS Secular changes in pediatric aerobic fitness test performance: the global picture Med Sport Sci 2007 50 46 66 full_text 17387251 10.1159/000101075

[B9] Shrewsbury V Wardle J Socioeconomic status and adiposity in childhood: a systematic review of cross-sectional studies 1990-2005 Obesity (Silver Spring) 2008 16 2 275 284 10.1038/oby.2007.35 18239633

[B10] Lasserre AM Chiolero A Cachat F Paccaud F Bovet P Overweight in Swiss children and associations with children's and parents' characteristics Obesity (Silver Spring) 2007 15 12 2912 2919 10.1038/oby.2007.347 18198298

[B11] Freitas D Maia J Beunen G Claessens A Thomis M Marques A Crespo M Lefevre J Socio-economic status, growth, physical activity and fitness: the Madeira Growth Study Ann Hum Biol 2007 34 1 107 122 10.1080/03014460601080983 17536760

[B12] Hawkins SS Cole TJ Law C Maternal employment and early childhood overweight: findings from the UK Millennium Cohort Study Int J Obes (Lond) 2008 32 1 30 38 10.1038/sj.ijo.0803682 17637703 PMC2679151

[B13] Janssen I Katzmarzyk PT Boyce WF Vereecken C Mulvihill C Roberts C Currie C Pickett W Comparison of overweight and obesity prevalence in school-aged youth from 34 countries and their relationships with physical activity and dietary patterns Obes Rev 2005 6 2 123 132 10.1111/j.1467-789X.2005.00176.x 15836463

[B14] Lobstein T Frelut ML Prevalence of overweight among children in Europe Obes Rev 2003 4 4 195 200 10.1046/j.1467-789X.2003.00116.x 14649370

[B15] Niederer I Kriemler S Zahner L Burgi F Ebenegger V Hartmann T Meyer U Schindler C Nydegger A Marques-Vidal P Influence of a lifestyle intervention in preschool children on physiological and psychological parameters (Ballabeina): study design of a cluster randomized controlled trial BMC Public Health 2009 9 94 19335890 10.1186/1471-2458-9-94 PMC2676270

[B16] Cole TJ Bellizzi MC Flegal KM Dietz WH Establishing a standard definition for child overweight and obesity worldwide: international survey BMJ 2000 320 7244 1240 1243 10.1136/bmj.320.7244.1240 10797032 PMC27365

[B17] Lohman TG Roche AF Martorell R Anthropometric Standardization Reference Manual 1988 Champaign: Human Kinetics Books

[B18] de Vries SI Bakker I Hopman-Rock M Hirasing RA van Mechelen W Clinimetric review of motion sensors in children and adolescents J Clin Epidemiol 2006 59 7 670 680 10.1016/j.jclinepi.2005.11.020 16765269

[B19] Pate RR Almeida MJ McIver KL Pfeiffer KA Dowda M Validation and calibration of an accelerometer in preschool children Obesity (Silver Spring) 2006 14 11 2000 2006 10.1038/oby.2006.234 17135617

[B20] Trost SG Pate RR Freedson PS Sallis JF Taylor WC Using objective physical activity measures with youth: how many days of monitoring are needed? Med Sci Sports Exerc 2000 32 2 426 431 10.1097/00005768-200002000-00025 10694127

[B21] Baquet G Stratton G Van Praagh E Berthoin S Improving physical activity assessment in prepubertal children with high-frequency accelerometry monitoring: a methodological issue Prev Med 2007 44 2 143 147 10.1016/j.ypmed.2006.10.004 17157370

[B22] Evenson KR Catellier DJ Gill K Ondrak KS McMurray RG Calibration of two objective measures of physical activity for children J Sports Sci 2008 26 14 1557 1565 10.1080/02640410802334196 18949660

[B23] Molnar D Livingstone B Physical activity in relation to overweight and obesity in children and adolescents Eur J Pediatr 2000 159 Suppl 1 S45 55 10.1007/PL00014365 11011955

[B24] Vogt U Die Motorik 3-bis 6jähriger Kinder : ihre Abhängigkeit vom biologischen Entwicklungsstand und sozialen Umweltfaktoren Diss Univ Berlin-West, 1976 1978 Verlag Karl Hofmann

[B25] Kunz T Weniger Unfälle durch Bewegung : mit Bewegungsspielen gegen Unfälle und Gesundheitsschäden bei Kindergartenkindern 1993 Schorndorf: Verlag Karl Hofmann

[B26] Faeh D Minder C Gutzwiller F Bopp M Culture, risk factors and mortality: can Switzerland add missing pieces to the European puzzle? J Epidemiol Community Health 2009 63 8 639 645 10.1136/jech.2008.081042 19386611

[B27] Sundblom E Petzold M Rasmussen F Callmer E Lissner L Childhood overweight and obesity prevalences levelling off in Stockholm but socioeconomic differences persist Int J Obes (Lond) 2008 32 10 1525 1530 10.1038/ijo.2008.104 18626485

[B28] Morgenstern M Sargent JD Hanewinkel R Relation between socioeconomic status and body mass index: evidence of an indirect path via television use Arch Pediatr Adolesc Med 2009 163 8 731 738 10.1001/archpediatrics.2009.78 19652105 PMC3719170

[B29] Caprio S Daniels SR Drewnowski A Kaufman FR Palinkas LA Rosenbloom AL Schwimmer JB Influence of race, ethnicity, and culture on childhood obesity: implications for prevention and treatment Obesity (Silver Spring) 2008 16 12 2566 2577 10.1038/oby.2008.398 19279654

[B30] Nelson MC Gordon-Larsen P Song Y Popkin BM Built and social environments associations with adolescent overweight and activity Am J Prev Med 2006 31 2 109 117 10.1016/j.amepre.2006.03.026 16829327

[B31] Cacciari E Milani S Balsamo A Dammacco F De Luca F Chiarelli F Pasquino AM Tonini G Vanelli M Italian cross-sectional growth charts for height, weight and BMI (6-20 y) Eur J Clin Nutr 2002 56 2 171 180 10.1038/sj.ejcn.1601314 11857051

[B32] Haug E Rasmussen M Samdal O Iannotti R Kelly C Borraccino A Vereecken C Melkevik O Lazzeri G Giacchi M Overweight in school-aged children and its relationship with demographic and lifestyle factors: results from the WHO-Collaborative Health Behaviour in School-aged Children (HBSC) study Int J Public Health 2009 54 Suppl 2 167 179 10.1007/s00038-009-5408-6 19618111 PMC2735089

[B33] Steptoe A Wardle J Fuller R Holte A Justo J Sanderman R Wichstrom L Leisure-time physical exercise: prevalence, attitudinal correlates, and behavioral correlates among young Europeans from 21 countries Prev Med 1997 26 6 845 854 10.1006/pmed.1997.0224 9388797

[B34] Lamprecht M Stamm HP Bewegung, Sport, Gesundheit - Fakten und Trend aus der Schweizerischen Gesundheitsbefragungen 1992, 1997, 2002 Stat santé - Resultate zu den Gesunheitsstatistiken in der Schweiz 2006 Neuchâtel: Bundesamt für Statistik (BFS)

[B35] Bringolf-Isler B Grize L Mader U Ruch N Sennhauser FH Braun-Fahrlander C Built environment, parents' perception, and children's vigorous outdoor play Prev Med 50 5-6 251 256 10.1016/j.ypmed.2010.03.008 20346370

[B36] Grize L Bringolf-Isler B Martin E Braun-Fahrlander C Trend in active transportation to school among Swiss school children and its associated factors: three cross-sectional surveys 1994, 2000 and 2005 Int J Behav Nutr Phys Act 7 28 10.1186/1479-5868-7-28 20398320 PMC2867987

[B37] Faeh D Bopp M Educational inequalities in mortality and associated risk factors: German- versus French-speaking Switzerland BMC Public Health 2010 10 1 567 10.1186/1471-2458-10-567 20858293 PMC2955004

[B38] Crawford D Cleland V Timperio A Salmon J Andrianopoulos N Roberts R Giles-Corti B Baur L Ball K The longitudinal influence of home and neighbourhood environments on children's body mass index and physical activity over 5 years: the CLAN study Int J Obes (Lond) 2010 34 7 1177 1187 10.1038/ijo.2010.57 20351728

[B39] Christakis NA Fowler JH The spread of obesity in a large social network over 32 years N Engl J Med 2007 357 4 370 379 10.1056/NEJMsa066082 17652652

[B40] Voorhees CC Murray D Welk G Birnbaum A Ribisl KM Johnson CC Pfeiffer KA Saksvig B Jobe JB The role of peer social network factors and physical activity in adolescent girls Am J Health Behav 2005 29 2 183 190 15698985 10.5993/ajhb.29.2.9 PMC2507875

